# High-accuracy detection of malaria vector larval habitats using drone-based multispectral imagery

**DOI:** 10.1371/journal.pntd.0007105

**Published:** 2019-01-17

**Authors:** Gabriel Carrasco-Escobar, Edgar Manrique, Jorge Ruiz-Cabrejos, Marlon Saavedra, Freddy Alava, Sara Bickersmith, Catharine Prussing, Joseph M. Vinetz, Jan E. Conn, Marta Moreno, Dionicia Gamboa

**Affiliations:** 1 Laboratorio ICEMR-Amazonia, Laboratorios de Investigación y Desarrollo, Facultad de Ciencias y Filosofía, Universidad Peruana Cayetano Heredia, Lima, Peru; 2 Facultad de Salud Pública, Universidad Peruana Cayetano Heredia, Lima, Peru; 3 Ministry of Health, Iquitos, Peru; 4 Wadsworth Center, New York State Department of Health, Albany, New York, United States of America; 5 Department of Biomedical Sciences, School of Public Health, State University of New York-Albany, Albany, New York, United States of America; 6 Division of Infectious Diseases, Department of Medicine, University of California San Diego, La Jolla, California, United States of America; 7 Instituto de Medicinal Tropical Alexander von Humboldt, Universidad Peruana Cayetano Heredia, Lima, Peru; 8 Departamento de Ciencias Celulares y Moleculares, Facultad de Ciencias y Filosofía, Universidad Peruana Cayetano Heredia, Lima, Peru; Institut de Recherche pour le Développement (IRD), FRANCE

## Abstract

Interest in larval source management (LSM) as an adjunct intervention to control and eliminate malaria transmission has recently increased mainly because long-lasting insecticidal nets (LLINs) and indoor residual spray (IRS) are ineffective against exophagic and exophilic mosquitoes. In Amazonian Peru, the identification of the most productive, positive water bodies would increase the impact of targeted mosquito control on aquatic life stages. The present study explores the use of unmanned aerial vehicles (drones) for identifying *Nyssorhynchus darlingi* (formerly *Anopheles darlingi)* breeding sites with high-resolution imagery (~0.02m/pixel) and their multispectral profile in Amazonian Peru. Our results show that high-resolution multispectral imagery can discriminate a profile of water bodies where *Ny*. *darlingi* is most likely to breed (overall accuracy 86.73%- 96.98%) with a moderate differentiation of spectral bands. This work provides proof-of-concept of the use of high-resolution images to detect malaria vector breeding sites in Amazonian Peru and such innovative methodology could be crucial for LSM malaria integrated interventions.

## Author summary

The most efficient malaria vector in the Latin American region is *Nyssorhynchus darlingi* (formerly *Anopheles darlingi*). In Amazonian Peru, where malaria is endemic, *Ny*. *darlingi* feeds both indoors and outdoors (endophagy, exophagy), depending on the local environment, and rests outdoors (exophily). LLINs and IRS, the most common tools employed for vector control, target endophagic and endophilic mosquitoes. Thus, they are only partially effective against *Ny*. *darlingi*. Control of the aquatic stages of vector mosquitoes, larval source management (LSM), targets the most productive breeding sites nearest to human habitation. In four riverine communities, we used drones with high-resolution imagery as a key initial step to analyze water bodies within the estimated flight range of *Ny*. *darlingi*, ~ 1 km. We found distinctive spectral profiles for water bodies that were positive *versus* negative for *Ny*. *darlingi*. The methodology and analysis reported here provide the basis for testing whether LSM can be combined successfully with LLINs and IRS to contribute to the elimination of transmission in malaria hotspots in the Amazon.

## Introduction

The most widespread strategies to combat malaria rely on the distribution of long-lasting insecticide-treated nets (LLINs) [1] and the application of indoor residual spray (IRS) [2] that target endophagic and endophilic mosquito vectors. The decline in their efficiency is associated mainly with: a) insecticide contact avoidance by early-exiting behavior of mosquitoes feeding indoors [3]; b) increased outdoor feeding and transmission; c) zoophilic behavior; and d) insecticide resistance [4]. Regional and local mosquito populations in Latin America frequently display both exophagic and exophilic feeding preferences, reducing the usefulness of these two widely-accepted strategies [5].

The urgent need to redesign vector control tools for mosquito populations resistant to current interventions has led to the targeting of key environmental resources, increasing the relevance of larval source management (LSM) [5–7]. Gravid female Anophelinae have the potential to discriminate among water bodies and seek suitable breeding sites for oviposition, using visual and olfactory cues [8]. Therefore, knowledge of the characterization and identification of the most productive, positive water bodies would help to increase the impact of targeted larval mosquito control. The current measures associated with LSM are oriented toward the use of larvicides and biological control. LSM trials have been conducted in Africa in part because the habitats of African anophelines are well characterized; such trials have shown that larvicides can reduce malaria transmission from 70–90% [7]. In the neotropics, the efficacy of larval control using *Bacillus sphaericus* against *Nyssorhynchus darlingi* (formerly *Anopheles darlingi* [9]) was evaluated in gold-mining pools [10] and in fish ponds [11] in the Brazilian Amazon. However, few studies have been performed in natural breeding sites [12, 13]. Two examples of studies highlighting successful larval control in natural breeding sites are one that employed *B*. *sphaericus* against *Nyssorhynchus aquasalis* in Venezuela in brackish mangroves [14] and another that implemented larvivorous nematodes in Colombia [15]. There are several impediments to identifying *Ny*. *darlingi* breeding sites in the Amazon basin. For example, potential breeding sites are periodically flooded, making field surveys difficult [16]; sometimes natural breeding sites are nearly impossible to detect visually by ground-truthing due to extensive, dense vegetation.

*Nyssorhynchus darlingi* is the primary malaria vector across the Amazon basin, accounting for up to 85% of the Anophelinae fauna feeding on humans [17–19]. This species is behaviorally very plastic, mainly biting and resting outdoors (exophily) with fewer reports of endophily (indoor resting; reviewed in [20]), and simultaneous endophagy and exophagy (reviewed in [21, 22]). In Amazonian Peru, there are regional records of both endo- and exophagy [17, 23], including behavioural shifts presumed to be in response to the implementation of LLINs [24]. In this region, mosquito abundance is linked to river levels [17, 25], which rise substantially during the rainy season, providing female mosquitoes with innumerable water bodies suitable for oviposition. However, in some specific situations, floods have been reported as one driver of *Ny*. *darlingi* population elimination [26].

*Nyssorhynchus darlingi* colonizes diverse water bodies, contributing to dispersal and diversification across its broad range, from natural areas [12, 13, 21, 22, 27, 28] to artificial (human-made) such as fish ponds, agricultural settlements, highways, mining sites and urban areas [29–31]. Sun exposure has been denoted as one of the determinant variables affecting oviposition site suitability, together with presence of water plants and secondary vegetation, green algae and reduced water current [12, 22, 28, 32].

Malaria transmission in the Peruvian Amazon is highly heterogeneous. Loreto Department (northeastern Peru) reports the vast majority (>95% of national cases; *e*.*g*. 53,163 of 55,210 in 2017) of the malaria cases in the country, with an estimated proportion of 80% *Plasmodium vivax* and 20% *P*. *falciparum* [33, 34]. However, there are areas punctuated by transmission pockets that account for most cases in the Department [34, 35]. Transmission occurs mainly during the rainy season, January—June, linked to river levels and mosquito abundance [23, 25, 36]. Parker and collaborators [37] demonstrated that high human biting rates (HBR), entomological inoculation rate (EIR), and infectivity of *Ny*. *darlingi* are a signature of remote riverine malaria hot spots and hyperendemicity in certain areas of the Peruvian Amazon, revising previous assumptions that transmission is hypoendemic throughout the peri-Iquitos region [17, 29, 38].

Classical survey techniques of larval habitats, in general, achieve small spatial coverage, limiting research on Anophelinae breeding sites, *i*.*e*., extended water bodies over large areas are not practical to survey from the ground due to the complex landscape and dynamic nature of such water bodies. Several studies have demonstrated the capability of satellite imagery to detect large *Ny*. *darlingi* breeding sites in several countries [39–41]. However, the spatial resolution of public (~30 meters/pixel) or private (~1 meter/pixel) satellite imagery is inadequate due to the high vegetation coverage and/or the quality of images related to climatic conditions in the Amazon Region, particularly during the extensive rainy season. Although there are applications for Unmanned Aerial Vehicles (UAVs a.k.a. drones) across many fields, such as monitoring crops [42] and forest [43], few researchers have taken advantage of this technology to investigate anopheline breeding sites linked to transmission pockets. Two recent studies have used UAVs to map land use and *Anopheles gambiae* breeding sites [44, 45] and to link malaria epidemiology with landscape ecology in Thailand [46]. Nevertheless, no parallel studies have been conducted in the Amazon Basin, which is operationally challenging with a considerable amount of potential Anophelinae larval habitat, especially during the rainy months.

The current study explores the use of drones for mapping water bodies in four rural villages in the Peruvian Amazon. Our main objective was to provide proof-of-concept of the suitability of high-resolution imagery (RGB band) to map *Ny*. *darlingi* aquatic habitats. Multi-spectral imaging data (including the normalized difference vegetation index- NDVI) was used to achieve sufficient resolution to identify water bodies potentially colonized by *Ny*. *darlingi*. The public health-oriented deployment of this approach to identify and target water bodies for use in LSM campaigns is discussed. The data here allow us to postulate that, in combination with existing vector interventions such as LLINs and IRS, drones could be an attractive additional tool for malaria elimination in the Amazon and other places where mosquito behavior and larval breeding sites remain difficult to locate and identify.

## Methods

### Ethics approval

Study protocols were approved by the Ethics Review Board of the Regional Health Directorate of Loreto (477–2016), Universidad Peruana Cayetano Heredia in Lima (184-09-16) and WHO Ethics Review Committee (0002669). These requirements were established by TDR/WHO despite the absence of human subject involvement in the present work. All the methods were carried out in accordance with the approved guidelines.

### Study areas

**Fig 1:**
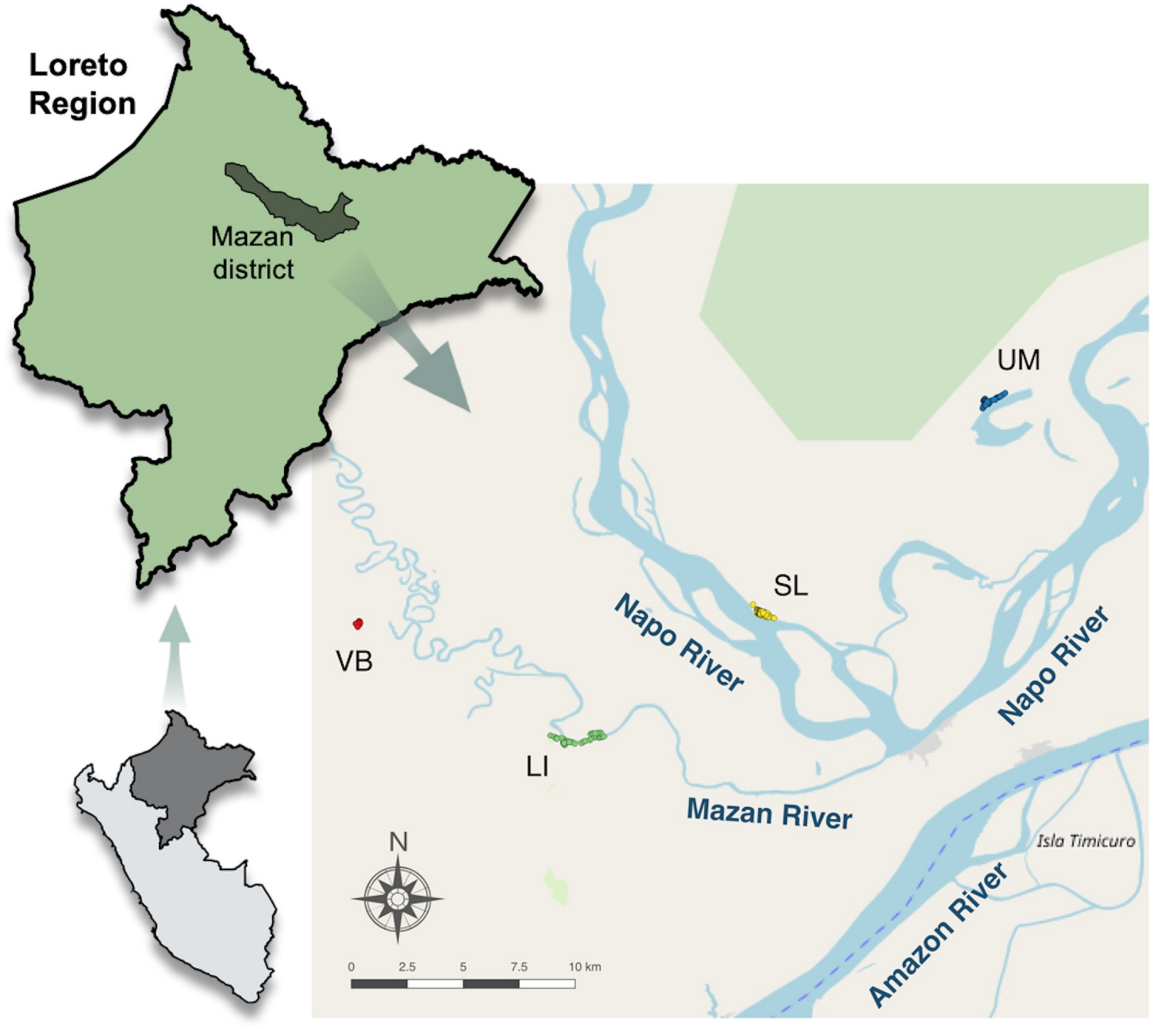
Study area in Mazan district, Loreto Region, Peruvian Amazon. Localization of Visto Bueno (VB, red dots), Libertad (LI, green dots), Salvador (SL, yellow dots) and Urco Miraño (UM, blue dots) communities in the Mazan district. Maps were produced using QGIS based on public geographic data obtained from OpenStreetMap (www.openstreetmap.org).

The study was conducted in the Mazan district (Maynas Province, Loreto Department, Peru) that has been identified as a very high-risk district for malaria transmission [47]. To represent as broadly as possible the landscape of this area, four communities were selected in two ecologically different river microbasins in the Mazan district [48]; two communities in the blackwater Mazan River district: Visto Bueno (3.449° S, 73.317° W; population = 60) and Libertad (3.496° S, 73.234° W; population = 345); and two in the whitewater Napo River: Salvador (3.445° S, 73.154° W; pop = 431) and Urco Miraño (3.361° S, 73.064° W; pop = 240). Map in Fig 1 was produced with QGIS 2.16 (QGIS Development Team, 2016. QGIS Geographic Information System. Open Source Geospatial Foundation Project) and based on public geographical data from OpenStreetMaps (www.openstreetmap.org). Detailed characteristics of these communities have been described elsewhere [47].

Mazan is a district in Loreto with sustained annual malaria transmission. The Regional Health Directorate of Loreto (RHDL) reported 1061 cases in 2016 caused mainly by *P*. *vivax* (68.5%) and *P*. *falciparum* (31.5%), equivalent to an Annual Parasite Index (API) of 78.9 cases per 1000 inhabitants. The RHDL passive case report is based exclusively on light microscopy and some studies demonstrate a large sub-microscopic malaria reservoir [16, 38]. In this area, a seasonal pattern of increase during the rainy season was observed in both malaria cases and vector abundance (predominantly *Ny*. *darlingi*) [25, 37].

### Study design

Drone surveys were carried out in the four communities between April 17 and 23, 2017. Mapping based on RGB and multispectral imagery was conducted simultaneously. In each community, water bodies were inspected at three time points—in September and November 2016 (dry season) and March 2017 (rainy season)—for the presence of *Ny*. *darlingi* immature stages; then, data from water bodies were available six months prior to the drone surveys.

### Data collection

#### Larval collections

To identify and characterize *Ny*. *darlingi* breeding sites, 31 water bodies–both artificial (i.e., fishponds) and natural (i.e., stream/creek, palm swamp)–located within 1km of each village reachable by ground inspection, were examined in the 4 communities of the Peruvian Amazon: 5 water bodies in Visto Bueno, 8 in Libertad, 9 in Salvador, and 9 in Urco Miraño (Fig 1). Larval sampling was performed using standard dippers (350 mL) with ten dips taken every 10 meters along the edge of the water body, with a maximum of 20 sampling locations per water body, to determine presence or absence of Anophelinae larvae. The same water bodies were sampled at each survey. All larval samples were preserved in 100% ethanol.

### Drone surveys

Drone surveys were carried out using a DJI Phantom 4 Pro (DJI, Shenzhen, China) quadcopter fitted with a DJI 4K camera (8.8 mm/24 mm; f/2.8; 1'' CMOS; 20 MP) for conventional RGB imagery collection and a 3DR Solo (3D Robotics, California, US) quadcopter fitted with a Parrot Sequoia sensor (Parrot, France) which is composed of single-band cameras (Green, Red, Red Edge and Near Infrared—nir) of 1.2 MP for multispectral imagery collection. The flight plan was programmed with Pix4D Capture app in an iPad Mini 4 (Apple, California, US). The connection between the controller and DJI Phantom 4 Pro and 3DR Solo was set up using DJI GO 4 app and 3DR Solo app, respectively.

For RGB mapping, in each community the DJI Phantom 4 Pro drone was flown to an altitude of approximately 100 m, which gave a ground sampling distance (GSD) or spatial resolution of 0.1 meter/pixel. Grids of 500m x 500m were drawn in Pix4D. Households and a buffer of at least 250m were covered using several grids in each community: 4 in Visto Bueno, 10 in Libertad, 9 in Salvador, and 8 in Urco Miraño. In each grid, 100 waypoints were automatically calculated to ensure an overlap of at least 70% between neighboring images, necessary to generate an orthomosaic [49]. The flight plan was preloaded onto the DJI Phantom 4 Pro drone and the flight path was followed automatically. A flying time of ~30 minutes without a change of battery was required to complete the survey in each grid.

Multispectral mapping was conducted over 16 randomly sampled water bodies (51.6% of water bodies inspected for *Ny*. *darlingi* larvae during the study), located as follows: 5 in Visto Bueno, 2 in Libertad, 4 in Salvador, and 5 in Urco Miraño. In each water body, the 3DR Solo drone was flown to an altitude of approximately 50m, which assured a GSD of 0.02 meter/pixel. A grid of 200m x 200m was drawn in Pix4D and the Sequoia multispectral camera was set up to take an image each second during the 20-minutes flight time of the 3DR Solo drone.

### Laboratory procedures

#### Larvae identification

All larvae were identified by species-specific ITS2 PCR-RFLP [50]; for the few samples that did not amplify, the mtDNA *COI* gene barcode region was sequenced [51] and compared with sequences available in GenBank or BOLD SYSTEMS v2.5 (http://www.barcodinglife.org) and the best match with identity of 95% or above was recorded. Only samples identified as *Ny*. *darlingi* were included in this study.

### Data processing

#### Orthomosaic construction

The photogrammetric processing (surface measurements based on photographs) was conducted in AgiSoft Photoscan Pro (https://www.agisoft.com). The resulting UAV imagery was imported into Photoscan and processed to construct an orthomosaic (georeferenced mosaic of overlapped images which includes correction for topographic distortions) for each community. The position of the drone at the time of image capture for each photo was recorded automatically by the on-board GPS; thus, an orthomosaic can be georeferenced without the need of Ground Control Points (GCP).

The standard procedure used was: (1) photo alignment (accuracy: highest; generic preselection active, reference preselection active; Key point limit: 80,000; adaptive camera model fitting active); (2) dense cloud building (quality: high; depth filtering: aggressive); (3) digital elevation model (DEM) building (geographic projection using WGS 84 (EPSG:4326); resolution of 0.1 m and 0.02 m per pixel for the RGB and multispectral images respectively; interpolation: extrapolated; all point classes to generate digital surface model); (4) orthomosaic building (input surface: DEM; blending mode: mosaic; resolution of 0.1 m and 0.02 m per pixel for the RGB and multispectral images respectively).

For each community, three orthomosaics were constructed: (1) a 3-band RGB image (Red, Green, and Blue) from the DJI 4K camera; (2) a 4-band multispectral image (Red, Green, Edge Red and Near Infrared) from the Parrot Sequoia camera; (3) an 8-band composite image (Table 1), merging the 3-band RGB and 4-band multispectral, plus a band of a normalized difference vegetation index (NDVI) calculated based on the bands from the Sequoia camera using the following formula:

$$NDVI=\frac{\left(NIR-Red\right)}{\left(NIR+Red\right)}$$

Due to the fact that multispectral imagery covers less area than RGB imagery, the 8-band composite was created using the areas where the orthomosaics intersected.

**Table 1:** RGB and multispectral bands used as features in the classification.

	Spectral band	label	Wavelength range	Resolution
**RGB Bands**	Blue	blue	492 to 455 nm	0.1 m
	Green	green	577 to 492 nm	
	Red	red	780 to 622 nm	
**Multispectral Bands**	Green	green_m	550 nm	0.02 m
	Red	red_m	660 nm	
	Red Edge	edge_red	735 nm	
	Near Infrared	nir	790 nm	
**Other**	NDVI	ndvi	-1 to 1	0.02 m

### Image classification

The image classification was conducted in Google Earth Engine (GEE) [52]. Briefly, GEE is a cloud-based platform for planetary-scale geospatial analysis that brings Google’s massive computational capabilities to bear on a variety of high-impact societal issues including deforestation, drought, disaster, disease, food security, water management, climate monitoring and environmental protection. It is unique in the field as an integrated platform designed to empower not only traditional remote sensing scientists, but also a much wider audience that lacks the technical capacity needed to utilize traditional supercomputers or large-scale commodity cloud computing resources [5].

All classification analyses were conducted in the online Integrated Development Environment (IDE) at https://code.earthengine.google.com (repositories for data and code available in Supplementary information). All 8-band multispectral orthomosaics were uploaded to GEE assets and a supervised classification was performed using a Random Forest (RF algorithm in GEE) [53]. RF is a collection of decision trees, also called CART (Classification and Regression trees) that has been widely used for mapping land cover in general. This method aims to associate specific targets with specific values of a particular variable; the result is a decision tree in which each part identifies a combination of values associated with a particular prediction [6]. The RF algorithm in GEE was set to 500 trees for each classification and was conducted using all bands in the 8-band orthomosaics as input. Default GEE parameters were used for the RF classification as follows: cross-validation factor for pruning = 10; maximal depth level of initial tree = 10; minimal leaf population = 1; minimal split population = 1; minimal split cost = 1e-10; whether to impose stopping criteria while growing the tree = false; quantization resolution for numerical feature = 100; quantization margin = 0.1.

RF classification use pre-labeled data as input. A dataset of polygons was constructed for each community in the study area, of which 480 were on-ground polygons and 240 were on-water polygons. Each class was composed of 30 samples per community, in total 120 samples per class. The total number of polygons per approach are presented in S1 Table Classes (or attributes) of on-ground polygons were labeled by *in situ* and ground inspection, whereas the on-water polygons classes were labeled using the results of the larvae sampling at the study area. For the classification, a water body was considered consistently positive if *Ny*. *darlingi* larvae were registered in 50% or more of the total visits and negative if *Ny*. *darlingi* larvae were recorded in less than 50% of the visits. In other words, if the water body was positive at least in 2 out of 3 or 1 out of 2 visits, the water body was considered consistently positive for *Ny*. *darlingi*.

Three approaches were used for the spatially explicit land cover classification: (1) a classifier with particular focus on identifying water bodies placing the orthomosaics into five groups: low vegetation, high vegetation, bare soil, urban and water bodies; (2) a classifier with a particular focus on differentiating water bodies with presence or absence of *Ny*. *darlingi* larvae, classifying the orthomosaics into six groups: low vegetation, high vegetation, bare soil, urban, water bodies positive for *Ny*. *darlingi* and water bodies negative for *Ny*. *darlingi*; and (3) a classifier with a particular focus on differentiating water bodies as positive or negative for *Ny*. *darlingi* classifying only the water bodies detected in approach 1 into two groups: water bodies positive and negative for *Ny*. *darlingi*. S1 Fig presents the workflow diagram of the three approaches.

### Training and validation

A *k*-fold cross validation was carried out to evaluate the performance of the RF classifier [54], thus, polygons served as training and validation samples. Briefly, all samples were randomly divided into *k* subsets (groups), for this study *k* was set to 5 (S2 Fig). The classifier was trained using four (*k*-1) groups and then tested with the remaining one. This procedure was repeated *k* times until all groups were used as a testing group. For each set of 4 training groups, the accuracy was calculated in the testing group. The mean accuracy of the *k* sets was considered as the overall accuracy (OA). In order to assess the probability distribution of the overall accuracy, the *k*-fold cross validation was repeated 999 times, where on each iteration a new random sample of polygons was assigned to each *k*-subset. Two additional performance measures were conducted, producer’s accuracy (PA), also called sensitivity, and consumer’s accuracy (CA), alternatively called positive predictive value (PPV).

In addition, to account for the spatial autocorrelation and lack of independence of polygons randomly selected at both training and test sets [55] a non-random groups assignment was conducted using the communities as natural groups (*k* = 4).

### Statistical analysis

In order to measure the statistical separability between positive (aquatic habitats consistently harboring *Ny*. *darlingi* >50% of the time)—and negative (aquatic habitats consistently harboring *Ny*. *darlingi* < 50% of the time)—water body classes in approaches 2 and 3, an interclass separability analysis was conducted using the Jeffries Matusita (JM) distance. Briefly, JM is a measure of the average difference between two-class (positive and negative water body) density functions by pair-wise comparison and ranges between 0 and 2 [56]. A JM distance of 0 imply no separation and 2 for full separation between land cover classes.

In addition, a Monte-Carlo coefficient/*p*-value/sample-size (CPS) sensitivity analysis was conducted. A complete description of the Monte-Carlo CPS is provided in the Supplementary Methods. All the implementations above were accomplished using R v.3.4.3 (R Development Core Ream, R Foundation for Statistical Computing, Australia).

## Results

### Mosquito breeding sites

From all water bodies inspected, 18 (58%) were considered negative and 13 (42%) consistently positive for the presence of *Ny*. *darlingi* immature stages. Of these, 16 (51.6%) were inside the mapped area of the 8-bands multispectral orthomosaics, and 8 were consistently positive for the presence of *Ny*. *darlingi* larvae. From the 16 water bodies sampled and multispectrally mapped, 4 (25%) provided information for only 2 of 3 collections because they were dry during 1 of the 3 visits, all of them in Visto Bueno. Importantly, none of the water bodies were dry during the drone survey. The proportion of water bodies positive for *Ny*. *darlingi* by community and survey is presented in S3 Fig for all water bodies inspected and for the 16 water bodies selected for multispectral mapping.

### Orthomosaics

**Fig 2:**
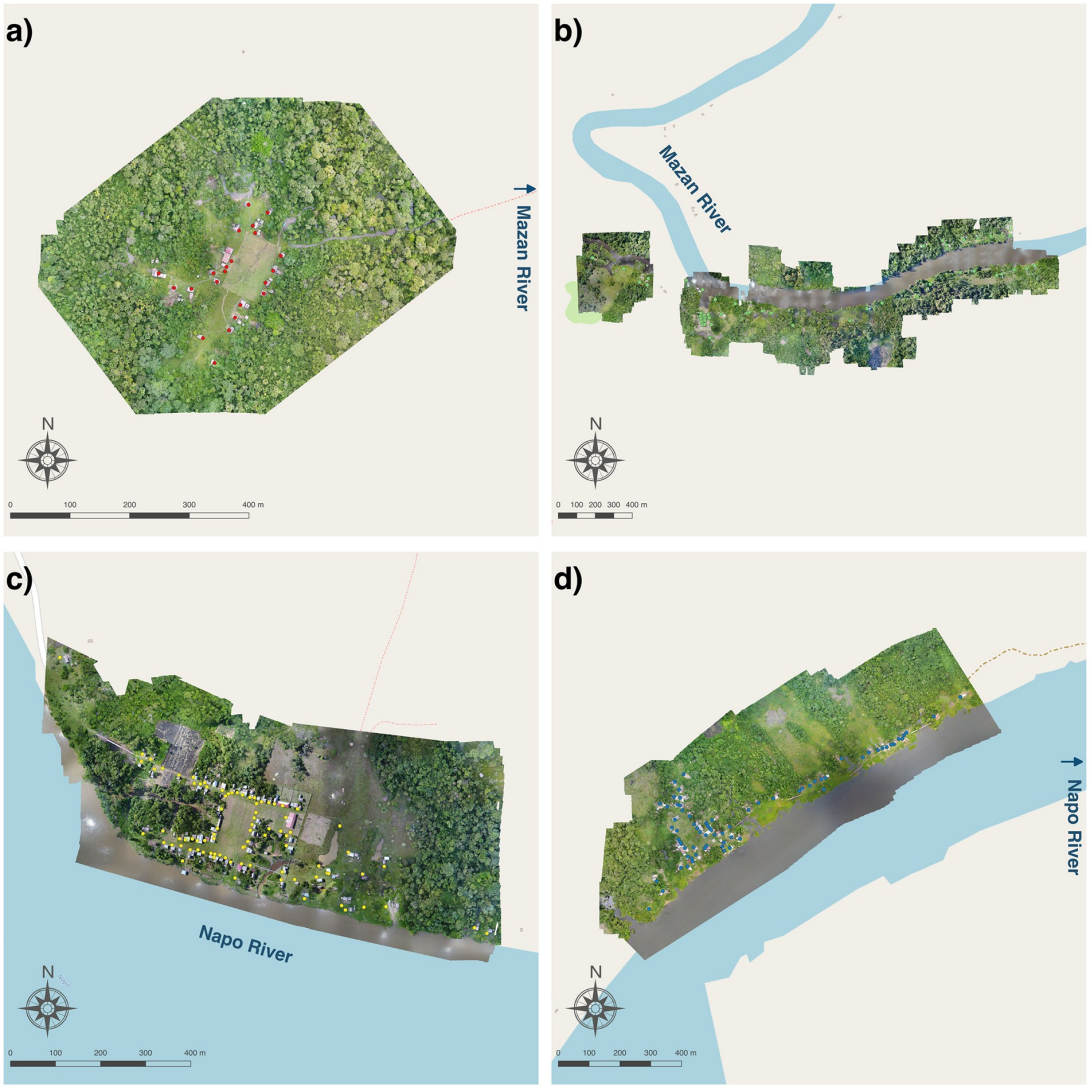
Orthomosaics of the communities of (a) Visto Bueno, (b) Libertad, (c) Salvador, (d) Urco Miraño. Orthomosaics were constructed in AgiSoft Photoscan Pro (https://www.agisoft.com)and mapped in QGIS. The basemaps were produced in QGIS based on public geographic data from OpenStreetMap (www.openstreetmap.org).

Several images were used to build the orthomosaic in each community. There were 386 RGB images in Visto Bueno, 1020 in Libertad, 805 in Salvador, and 958 in Urco Miraño; and there were 3804 Multispectral images in Visto Bueno, 7080 in Libertad, 6980 in Salvador, and 6940 in Urco Miraño (note that Parrot Sequoia captures 4 individual spectral band images per shot). An orthomosaic for each community is presented in Fig 2, and the 3D models in S4 Fig. The high spatial resolution of the resulting orthomosaics allowed for a clear identification of water bodies via simple visual inspection. However, is important to notice the limitations of the Structure-From-Motion algorithm (SfM) in Photoscan to match points in complex canopy environments where there is too much texture, poor illumination, and/or insufficient unique features, resulting in some gaps observed in S4 Fig [57].

**Fig 3:**
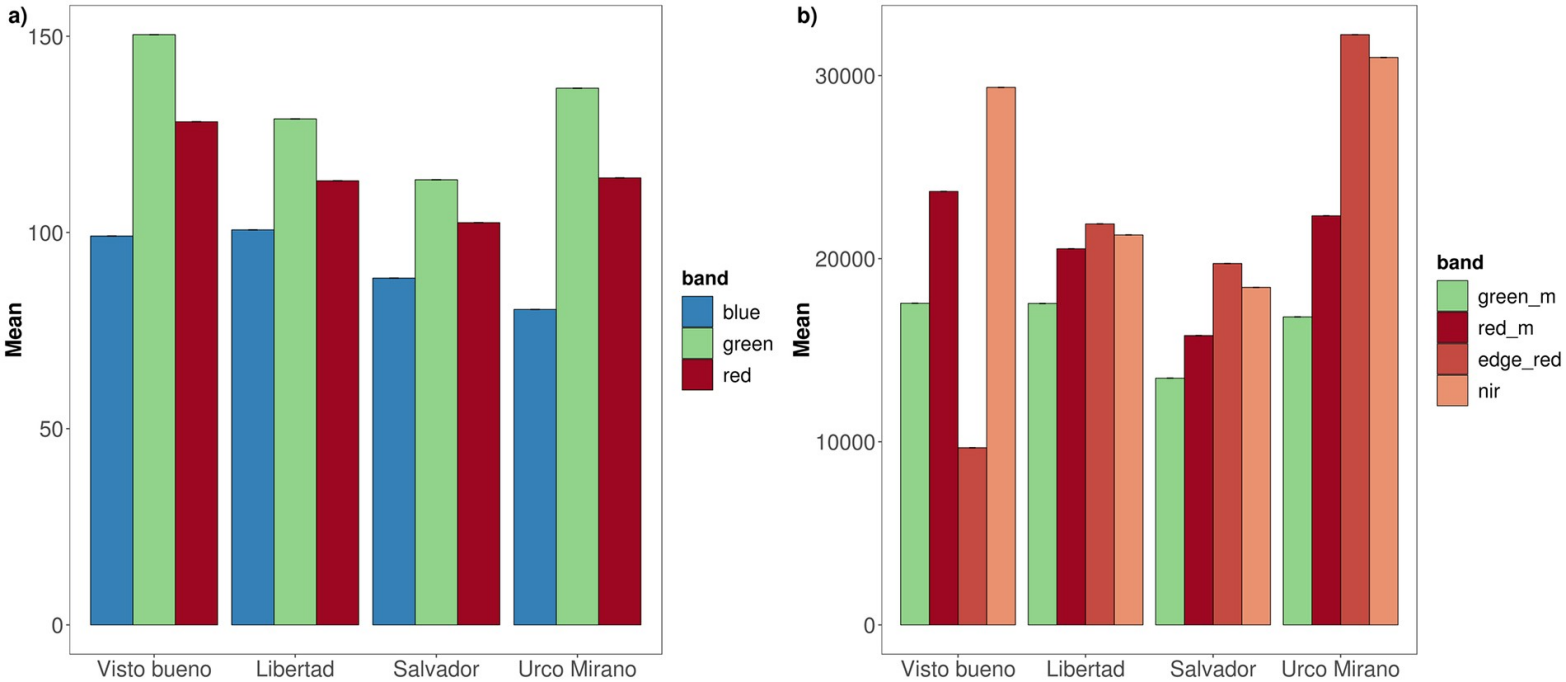
Mean values of bands in each community for a) RGB imagery (8-bit; blue, green, red) and b) Multispectral imagery (16-bit; green_m: Green, red_m: Red, edge_red: Edge Red, nir: Near Infrared).

**Fig 4:**
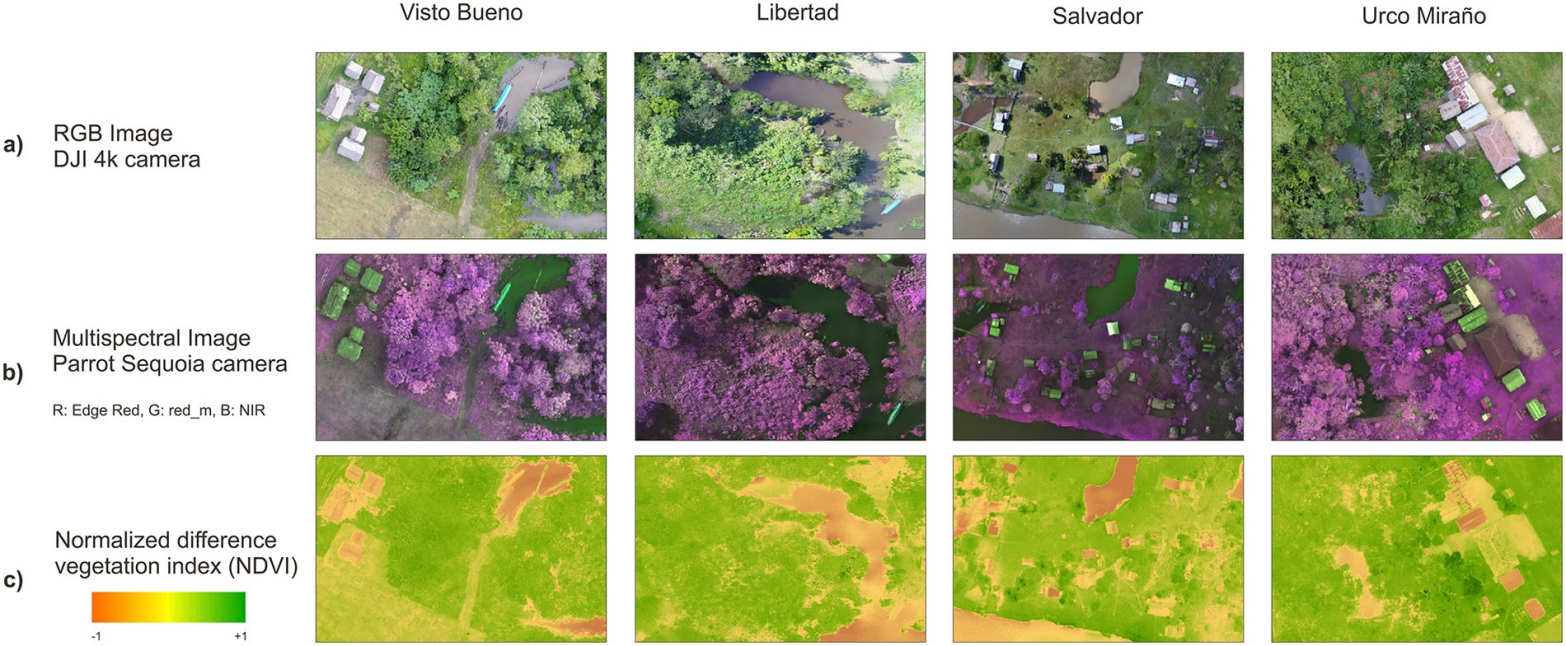
Orthomosaic scenes of the communities of Visto Bueno, Libertad, Salvador, and Urco Miraño using a) RGB imagery, b) Multispectral imagery, and c) Normalized Difference Vegetation Index (NDVI). Orthomosaics based on drone imagery were constructed in AgiSoft Photoscan Pro (https://www.agisoft.com)and mapped in QGIS.

Mean values and the standard errors for each band at each community are presented in Table 2; the RGB bands values are presented in 8-bit and the multispectral bands are in 16-bit. A heterogeneous spectral profile was observed between communities (Fig 3), presumably due to different environment and land cover composition. An example of a landscape using RGB, Multispectral and NDVI for each community is presented in Fig 4.

**Table 2:** Mean and standard error of RGB and multispectral bands in each community.

Bands		RGB bands (8-bit)			Multispectral bands (16-bit)				Other
Communities		blue	green	red	green_m	red_m	edge_red	nir	NDVI
Visto Bueno	Mean	99.08	150.4	128.2	17564.94	23671.37	9674.06	29354.12	0.1075
	Std. Error	0.0033	0.0033	0.0037	0.6117	0.7461	0.9048	0.8512	0
Libertad	Mean	100.66	128.93	113.15	17553.14	20541.31	21898.25	21294.79	0.0228
	Std. Error	0.0047	0.0052	0.0054	0.7859	0.8988	1.2812	1.2892	0
Salvador	Mean	88.36	113.41	102.49	13473.59	15800.67	19734.98	18432.08	0.0561
	Std. Error	0.0034	0.0027	0.0033	0.4981	0.4427	0.6576	0.6242	0
Urco Miraño	Mean	80.38	136.76	113.9	16818.42	22341.06	32233.27	30987.28	0.1576
	Std. Error	0.0027	0.0025	0.0028	0.4602	0.5068	0.6573	0.6224	0

### Random forest classification and validation

**Fig 5:**
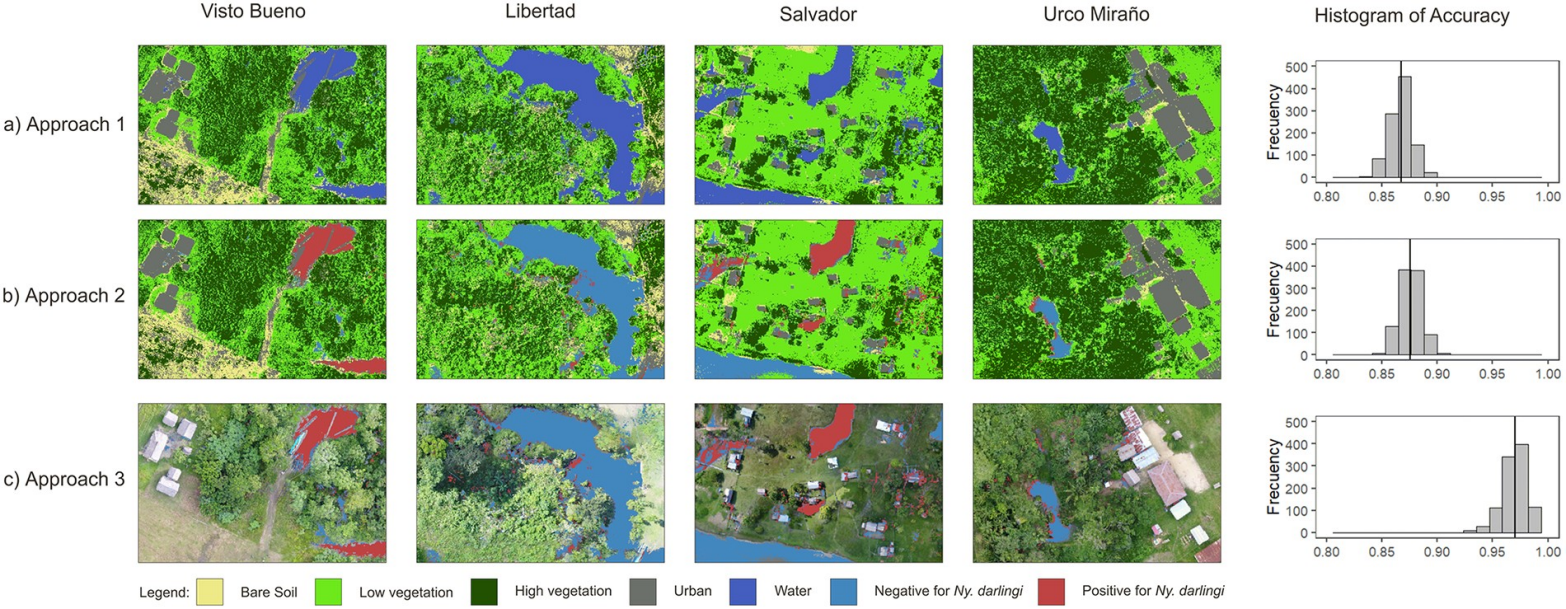
Output of the RF classification and accuracy distribution using 999 iterations of the k-fold validation for the communities of Visto Bueno, Libertad, Salvador, and Urco Miraño using: A) Approach 1, b) Approach 2, c) Approach 3. The basemap were orthomosaics based on drone imagery constructed in AgiSoft Photoscan Pro (https://www.agisoft.com)and mapped in QGIS.

**Fig 6:**
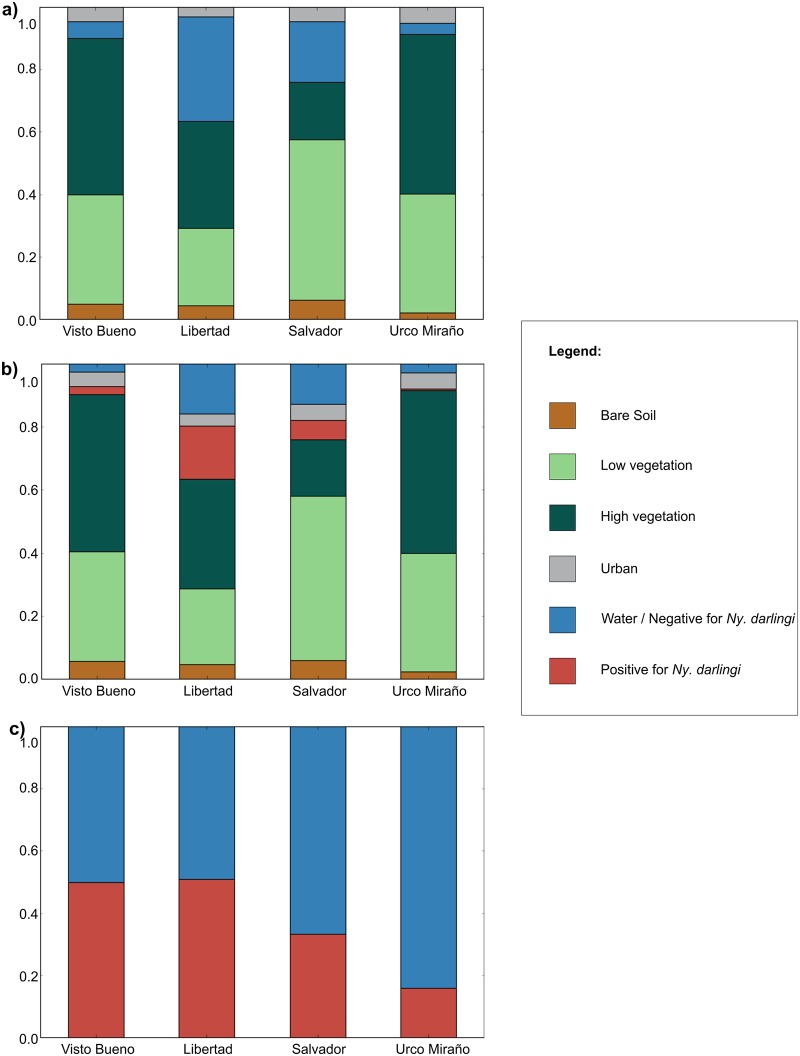
Proportion of land cover in each community classified by a) approach 1, b) approach 2, c) approach 3 of the Random Forest classification with random groups assignment.

Three approaches were used for the spatially explicit land cover classification in Google Earth Engine (GEE). The classified images for each community using the first approach are presented in Fig 5a. This approach showed high accuracy for differentiating among 4 land cover classes (bare soil, low- and high- vegetation, and urban) and water bodies. After 999 iterations, the overall accuracy of approach 1 was 86.73% (SE = 0.031). Classification approach 2 includes the differentiation of water bodies based on the presence of *Ny*. *darlingi* in the previous 6 months, in addition to the 4 land cover classes used in approach 1, with an overall accuracy of 87.58% (SE = 0.029) (Fig 5b). In approach 3, the 8-band composite image was masked using the water class obtained in approach 1. This approach shows the highest overall accuracy, with an average of 96.98% (SE = 0.025) (Fig 5c). The three approaches consistently depict highly heterogeneous land cover composition among the communities in the study (Fig 6). As these communities are located in the same district, this may reflect a high diversity of locations at the microgeographical scale where *Ny*. *darlingi* can breed.

Regarding the classification with non-random subsets, using communities as natural groups, this resulted in a diminished overall accuracy for approach 1 and 2 (63.92% and 65.70%, respectively). However, approach 3 still showed a high overall accuracy (92.26%). The overall accuracy of random and non-random assignment cross-validations is presented in Table 3; producer and consumer accuracies of each class are presented in S2 Table for random assignment and S3 Table for non-random assignment.

**Table 3:** Overall Accuracy of random and non-random assignment cross-validation for approach 1, approach 2 and approach 3.

	Approach 1	Approach 2	Approach 3
**Random assignment (k = 5)**			
Overall Accuracy	86.73%	87.58%	96.98%
Standard Error	0.031%	0.029%	0.025%
**No-random assignment (k = 4)**			
Visto Bueno	54.66%	56.32%	85.19%
Libertad	68.78%	69.82%	90.16%
Salvador	63.39%	67.71%	93.67%
Urco Miraño	68.85%	68.95%	100.00%
Overall Accuracy	63.92%	65.70%	92.26%

### Statistical analysis of spectral profile of water bodies

In approach 2, the resulting number of pixels classified as positive water bodies was 31'717,931 and 44'391,373 pixels for negative water bodies. A higher number of pixels was included in the analysis of approach 3, 35'211,614 for positive and 46'894,706 for negative water bodies. The mean, standard deviation and comparison of each band are shown in Table 4 for both approaches.

**Table 4:** Spectral profile differences between water bodies positive and negative for *Ny*. *darlingi* in classification approach 2 and 3.

Bands	Approach 2					Approach 3				
	Positive (n = 31'717,931)		Negative (n = 44'391,373)		p-val	Positive (n = 35'211,614)		Negative (n = 46'894,706)		p-val
	mean	sd	Mean	sd		mean	sd	mean	sd	
blue	100.647	44.38	107.094	38.162	<0.001	105.894	41.703	120.452	30.233	<0.001
green	119.193	36.188	125.391	34.017	<0.001	114.104	38.029	127.993	28.282	<0.001
red	114.875	40.246	116.538	39.571	<0.001	109.504	42.959	128.869	29.267	<0.001
green_m	19588.468	6902.937	15691.39	4646.648	<0.001	19752.087	7434.394	15975.805	4489.076	<0.001
red_m	19929.123	6676.902	15331.156	4969.932	<0.001	18739.104	6583.356	14515.692	3091.811	<0.001
edge_red	13652.695	8414.74	13117.885	8111.416	<0.001	11229.354	3325.798	11320.756	4325.616	<0.001
nir	12208.338	8404.298	11712.544	7788.674	<0.001	9551.923	1910.505	9431.09	2220.418	<0.001
ndvi	-0.262	0.241	-0.171	0.187	<0.001	-0.291	0.205	-0.21	0.151	<0.001

Overall, JM distances of each band between positive and negative water body classes are very low. The highest values of JM were shown in green_m and red_m bands in both approaches (Table 5). Consistently, Monte-Carlo CPS sensitivity analysis show that bands green_m, red_m, but also NDVI, show a noteworthy effect size for approach 2. Green_m and red_m show increased values in positive water bodies whereas higher values of NDVI were observed in negative water bodies. Interestingly, all bands except edge_red and nir were statistically meaningful in approach 3. The bands that showed increased values in positive water bodies are green_m and red_m. Conversely, blue, green, red, and NDVI bands showed higher values in negative water bodies (S5 and S6 Figs).

**Table 5:** Jeffries Matusita (JM) distance of each bands between positive- and negative- water body classes in approach 2 and 3.

	Approach 2	Approach 3
blue	0.0114	0.0781
green	0.0032	0.0769
red	0.0036	0.1504
green_m	0.3249	0.3824
red_m	0.3382	0.4849
edge_red	0.0639	0.0680
nir	0.1299	0.0188
ndvi	0.1063	0.1265

## Discussion

The present study is the first that explores the use of drone-based high-resolution mapping of *Ny*. *darlingi* breeding sites in the Amazon region. Both RBG and multispectral imagery were successfully acquired, allowing the analysis of a greater number of water bodies than ground field inspection, as well as the determination of local characteristics of *Ny*. *darlingi* habitats. Overall, the most important result of this study is the accurate classification of water bodies that enables discrimination between those that are consistently colonized by *Ny*. *darlingi* immature stages and those that are not. We believe that this strategy represents a new tool for tailored interventions for control and surveillance of malaria transmission in rural communities of the Peruvian Amazon and elsewhere.

### Successful mapping of *Nyssorhynchus darlingi* aquatic habitats

Difficulty in identifying and detecting *Ny*. *darlingi* breeding sites arises from vast and often difficult to access places where this species can successfully breed. The portability of UAVs allows investigators to navigate moderately hostile and complex environments, such as the Amazon Basin. This study assessed the feasibility of using UAVs to generate maps with a higher resolution compared to those available through satellites, mainly when the imagery required is specific to a local scale within a community or limited area of interest at a microgeographical scale. Previous studies also propose the use of UAV for mapping environmental risk factors for zoonotic malaria in Malaysia and Philippines [44], and vector habitats in Zanzibar [45]. The current study proved that in addition to RGB imagery, multispectral imagery collection is also feasible in rural areas, and the addition of this information boosted the distinction of environmental characteristics of water bodies that harbor *Ny*. *darlingi* larvae [58–60]. Capturing data multiple times in longitudinal entomological surveys potentially would provide the tools to study Anophelinae breeding site dynamics [45]. For instance, the adaptation to more permanent anthropogenic larval habitats has been hypothesized to be the cause of a resident population of *Ny*. *darlingi* in Porto Velho (Brazil), leading to a perennial presence of this species and probably promoting and maintaining continual *Plasmodium* transmission [61].

### Accurate classification of *Nyssorhynchus darlingi* breeding sites

The data reported here classified *Ny*. *darlingi* -positive and -negative water bodies. A high concordance of location and extent of water bodies was observed in the three approaches applied. An average accuracy between 87% and 97% with a relatively narrow distribution demonstrates a valid strategy to identify and prioritize water bodies for outdoor interventions such as LSM [62], microbial larvicides [63], or attractive toxic sugar baits (ATSBs) [64]. As the implementation of this classifier harnessed Google’s cloud-computing platform, a short length of time is required to complete the classification, overcoming computing resource limitations [65, 66].

### Environmental modifications

Modifications of ecosystems and natural resources frequently contribute to the emergence and spread of infectious disease agents. Specifically, land use changes including deforestation, irrigation, wetland modification and road construction, among others, have been identified as major drivers of infectious disease outbreaks and also can interfere in their transmission dynamics [67]. Malaria has been associated with these anthropogenic alterations in Asia [68], Africa [69] and Latin America [70] and of special concern is the creation of new breeding sites that may be increasing the proliferation of mosquitoes [71]. For example, *Ny*. *darlingi* uses a range of natural and artificial sites for breeding and is able to exploit highly diverse habitats [22, 26, 72] including deforested areas with substantial surrounding vertical vegetation [29, 41, 73]. Recently, fish farming has been promoted as a way to increase economic opportunity in rural localities in Brazil and Peru, and throughout Latin America. Unfortunately, these fishponds also provide ideal breeding sites for *Ny*. *darlingi* (holding 4-fold more Anophelinae larvae than natural water bodies), demonstrating a rapid adaptability to some new environmental niches, associated with concomitant increases in malaria case numbers, e.g., in Mancio Lima, Acre state, Brazil and along the Iquitos/Nauta highway, Loreto, Peru [41, 74].

The use of imagery acquired from drones may be helpful for the detection of landscape modifications in a rapidly changing environment that can affect mosquito population distribution. A recent study described distinct *Ny*. *darlingi* populations related to urban or rural settlements in Acre, Brazil with different grades of anthropogenic landscape modification [75]. Here, deforestation was the most plausible cause for loss of genetic diversity in the mosquito populations. Modifications in landscape affects physicochemical characteristics and/or ecological communities of Anophelinae breeding sites and this also may affect malaria transmission dynamics. For instance, *Plasmodium* transmission potential, including survival and extrinsic incubation period, has been demonstrated to be affected by larval food quantity in *Anopheles stephensi* [76]. Furthermore, *Anopheles coluzzii* has different permissiveness to *Plasmodium* depending on the nature of the diet associated with microbiota composition [77].

### Land cover differentiation between study sites

The findings in this study suggest strong differential microenvironmental composition of *Ny*. *darlingi* breeding sites compared with other less favorable water bodies that could be assessed with the combination of RGB and multispectral imagery. These differences were evaluated by the inspection of certain bands of the spectral profile between communities and the resulting land cover classification discussed above. As these patterns were observed in four communities in two microbasins of the Amazon region, these findings may be generalizable in similar contexts elsewhere and denote heterogeneous environmental characteristics at a microgeographical scale [78]. As discussed previously, *Ny*. *darlingi* dominates all these diverse microhabitats in the communities under study. Moreover, *Parker et*. *al*. [37] reported that *An*. *darlingi* comprised the majority of the mosquitoes collected in 21 sites along approximately 100 km of the Mazan river microbasin.

### Link with epidemiology

Knowledge of rapidly changing patterns of human settlements and vector distribution is vital for predicting disease risks and effectively targeting disease control measures. Interestingly, Libertad and Urco Miraño, the sites with the highest and the lowest proportion of area of water bodies with *Ny*. *darlingi* larvae, were reported as communities with very high and low malaria transmission, respectively [47]. In addition, the high heterogeneity in malaria incidence [47] reported in the Mazan and Napo river microbasins may have arisen in part from the highly heterogeneous environmental composition of each community and the productivity of Anophelinae in these habitat types [79]. Considering that this study was not designed to demonstrate any association between malaria risk and microhabitat composition of *Ny*. *darlingi*, further research is needed to obtain a time-series of high-resolution imagery to detect fluctuations in the spectral profile of aquatic habitats, leading to the development of accurate risk maps and to the identification of potential effects on subsequent local malaria transmission.

### Other applications

Drone-based mapping could have a wider range of applications. For instance, high resolution digital elevation models (DEM) are useful tools to analyze watersheds and small streams [80, 81], favorable to *Ny*. *darlingi* breeding sites that are shaped by intermittent heavy rain [82] [83, 84]. Moreover, these DEMs support the identification of seasonally flooded areas, common in the Amazon basin, that possibly increase human-mosquito contact and therefore are associated with a higher risk of malaria [82, 85–87]. Importantly, canopy coverage prevents DEM reconstruction in forested areas due to SfM photogrammetric issues, in consequence DEM must rely on other sensors such as Laser Imaging Detection and Ranging (LIDAR), that are more expensive and logistically demanding. However, photogrammetric-based DEM could still be useful for localized characterization of terrain in the forest fringes where *Ny*. *darlingi* demonstrates a breeding site preference in rural Amazon, [29, 41, 73].

In 2011 the Amazon river (and tributaries in Iquitos, Peru) experienced an unusual flooding event, a peak of the river level over 10 m, most likely associated with climatic events (El Nino Southern Oscillation-ENSO), altering the temporality and characteristics of water bodies and resulting in a replacement event of *Ny*. *darlingi* populations [88]. In Surinam, abnormal flooding of rivers with subsequent inundation of larval habitats was reported as one of the factors that destroyed a local *Ny*. *darlingi* population (together with ITN distribution and other interventions) [26].

Another key benefit of the UAVs for high-resolution mapping is the rapid assessment of house positions. This approach offers the opportunity to pinpoint the GPS coordinates of several human dwellings with a high accuracy in a single flight path, rather than the more laborious ground inspection of each dwelling. Also, this technology can help epidemiologists to understand spatial malaria transmission and human travel patterns [47].

The present study showed that in addition to traditional RGB mapping, multispectral bands add critical information to differentiate water bodies (independently, whether or not they harbor *Ny*. *darlingi* larvae), and other types of land cover in the Amazon Region. A limited set of low-cost cameras and drones were tested, therefore an evaluation of a wider range of commercially available options is recommended. Despite initial capital cost, scaling up of drone flights in multiple settings and times would require small investments. It is important to note the limitation of the extent of covered area with drone flights due to energy consumption; if large areas are required to be covered in a short time period, multiple drones would be necessary, increasing the cost of this implementation. Importantly, due to the abundance, tangled distribution, and unclear boundaries of the water bodies in the Amazon Region, the classification approach showed in this study could be preferred over manually delineation demonstrated in other settings [45]. The computing time of a single classification in GEE is less than a minute, however training and test sets are only applicable to the Peruvian Amazon Region. The addition of training and test sets of contrasting locations should be included to test transferability to a variety of scenarios.

### Limitations

We recognize some potential shortcomings in this study. The equipment used was of the highest quality and lowest price on the market at the time of the field study; this strategy would be more cost-effective as the number of surveys increase. To overcome this, several projects in other fields are proposing to utilize low-cost non-commercial UAVs that may help to spread the strategy [89, 90]. Another caveat is the limited flight time of the drone. Thus, several flights over the locality are required to obtain a single map, and may represent some deviation in the time between scenes of the unique map, with a potential effect on the spectral signature, although this is likely relatively minor. Also, the flight of a UAV requires a certain degree of expertise, however, steps in flight path automation will overcome this difficulty [91, 92]. Despite our use of the recommended overlap percentage in this study, some gaps in final imagery through forest canopy and some water bodies, as observed in S4 Fig, may have affected the final classification.

Regrettably, land cover classification using Google Earth Engine depends on an internet connection. With this in mind, transport of imagery in any physical storage unit to a point with a stable internet connection is feasible; however, the number and cost of storage units should be taken into account. Because this methodology is at an early stage, there is a lack of methods for rapid data processing and developing strategies to speed up image processing methods, but these are currently expanding. Multispectral camera calibration and climatic conditions, such as heavy rain, may also jeopardize imagery collection. However, in this study all flights were conducted during the same time range (over a few days) and under low cloud coverage and wind conditions to reduce the effect on the spectral signature. Overall the most important methodological caveat in this study is the definition of negative water bodies. Although we sampled only 8 negative and 8 positive water bodies for the presence of *Ny*. *darlingi*, the water body type included streams, fishponds and palm swamps and we sampled in two distinctive river microbasins. As this is a proof-of-concept study, future work should consider more frequent surveillance of these and additional water bodies from more communities and additional flights over the survey localities at different times of the day and under various atmospheric conditions.

### Conclusions

In summary, the use of high-resolution imagery can provide a better understanding of environment-related disease changes and can play a meaningful part in the development of decision-support tools. Our findings back the use of a low-cost UAVs and a freely available planetary cloud-based platform to achieve a highly accurate classification of the differential spectral signature of water bodies that harbor *Ny*. *darlingi* larvae and those that do not, in the Amazon region. This strategy might be generalizable to similar contexts elsewhere, resulting in new ways to control and survey malaria in affected settings, in combination with existing approaches.

## Supporting information

S1 Methods Monte-Carlo coefficient/p-value/sample-size (CPS) sensitivity analysis. (DOCX)

S1 Table Number of polygons in each classification approach. (DOCX)

S2 Table Producer and Consumer accuracies of random groups for approach 1, approach 2 and approach 3. (DOCX)

S3 Table Producer and Consumer accuracies of non-random groups for approach 1, approach 2 and approach 3. (DOCX)

S1 Fig Workflow of the three classification approaches. (TIF)

S2 Fig K-fold diagram. (TIF)

S3 Fig Proportion of water bodies with presence of *Ny*. *darlingi* by community and survey. a) All water bodies sampled and b) 16 water bodies selected for multispectral mapping.
(TIF)

S4 Fig 3D models based on DEM of the communities of (a) Visto Bueno, (b) Libertad, (c) Salvador, (d) Urco Miraño. 3D models were constructed and mapped in AgiSoft Photoscan Pro (https://www.agisoft.com)based on drone imagery.
(TIF)

S5 Fig Monte-Carlo CPS chart for classification approach 2. a) Standardized effect size and b) p-value as a function of sample size.
(TIF)

S6 Fig Monte-Carlo CPS chart for classification approach 3. a) Standardized effect size and b) p-value as a function of sample size.
(TIF)

S1 Data Data and code availability. (DOCX)
