# Supplementary material for: High-accuracy detection of malaria vector larval habitats using drone-based multispectral imagery
Source: PLoS Negl Trop Dis. 2019 Jan 17;13(1):e0007105. doi: 10.1371/journal.pntd.0007105 (PMC6353212; doi:10.1371/journal.pntd.0007105)
Supplement: S1 Methods — (DOCX) [file pntd.0007105.s001.docx]

# SUPPLEMENTARY METHODS

**Monte-Carlo coefficient/*p*-value/sample-size (CPS) sensitivity analysis:** The value of each pixel was extracted and classified accordingly to positive or negative water body classes from approaches 2 and 3. An unequal variances *t-*test (Welch's *t*-test) was used to compare the mean of each band between water bodies positive and negative for *Ny. darlingi*. A high number of pixels are expected, and the possible pitfalls (false positive signals) of the increased power due to the large data set in this analysis has been previously described [8]. To address this concern, a sensitivity analysis was conducted using a Monte-Carlo coefficient/*p*-value/sample-size (CPS) chart in order to overcome the *p*-value problem due to large sample size by plotting the effect size and *p*-value as a function of the sample size [55, 56]. Briefly, the Monte-Carlo CPS chart is based on repeatedly drawing multiple samples of increasing sizes, and then rerunning the analysis in each sample to construct the distribution of the sample statistics. As RGB and Multispectral imagery were collected with different scales, 8- and 16- bit respectively, in each sample we computed the standardized effect size using *Cohen’s d* formula [7]:

$$\boldsymbol{Effect size=}\frac{\boldsymbol{mean (positive)}\boldsymbol{-}\boldsymbol{mean (negative)}}{\boldsymbol{SD}_{\boldsymbol{pooled}}}$$

The pooled standard deviation (SD_pooled_) was calculated using the following formula:

$$\boldsymbol{SD}_{\boldsymbol{pooled}}\boldsymbol{=}\sqrt{\frac{\left( \boldsymbol{SD}_{\boldsymbol{positive}}^{\boldsymbol{2}}\boldsymbol{+}\boldsymbol{SD}_{\boldsymbol{negative}}^{\boldsymbol{2}} \right)}{\boldsymbol{2}}}$$

The distributions of the effect size and Welch's *t*-test *p*-value of 400 samples were plotted for each sample size, for a set of increasing sample sizes from 100 to 2000, increasing by 100 each time.
