# Supplementary material for: High-accuracy detection of malaria vector larval habitats using drone-based multispectral imagery
Source: PLoS Negl Trop Dis. 2019 Jan 17;13(1):e0007105. doi: 10.1371/journal.pntd.0007105 (PMC6353212; doi:10.1371/journal.pntd.0007105)
Supplement: S1 Table — (DOCX) [file pntd.0007105.s002.docx]

**Supplementary Table 1**: Number of polygons in each classification approach.

|  | Approach 1 | Approach 2 | Approach 3 |
| --- | --- | --- | --- |
| Low vegetation | 120 | 120 |  |
| High vegetation | 120 | 120 |  |
| Bare soil | 120 | 120 |  |
| Urban | 120 | 120 |  |
| Water body | 120 |  |  |
| Positive for *Ny. darlingi* | | 120 | 120 |
| Negative for *Ny. darlingi* | | 120 | 120 |
| Total polygons | 600 | 720 | 240 |
