# Supplementary material for: High-accuracy detection of malaria vector larval habitats using drone-based multispectral imagery
Source: PLoS Negl Trop Dis. 2019 Jan 17;13(1):e0007105. doi: 10.1371/journal.pntd.0007105 (PMC6353212; doi:10.1371/journal.pntd.0007105)
Supplement: S2 Table — (DOCX) [file pntd.0007105.s003.docx]

# Supplementary Table 2: Producer and Consumer accuracies of random groups for approach 1, approach 2 and approach 3.

|  | **Consumer Accuracy** | |  | **Producer Accuracy** | |
| --- | --- | --- | --- | --- | --- |
| **Classes** | **Mean** | **SE** |  | **Mean** | **SE** |
| **Approach 1** |  |  |  |  |  |
| Low Vegetation | 76.16% | 0.41% |  | 52.23% | 0.25% |
| High Vegetation | 83.84% | 0.08% |  | 83.88% | 0.08% |
| Bare Soil | 84.54% | 0.07% |  | 85.19% | 0.08% |
| Urban | 97.82% | 0.02% |  | 98.63% | 0.01% |
| Water Body | 93.47% | 0.04% |  | 97.92% | 0.03% |
| **Approach 2** |  |  |  |  |  |
| Low Vegetation | 77.64% | 0.20% |  | 68.90% | 0.17% |
| High Vegetation | 76.21% | 0.10% |  | 71.95% | 0.10% |
| Bare Soil | 86.90% | 0.05% |  | 89.15% | 0.06% |
| Urban | 90.66% | 0.06% |  | 92.80% | 0.07% |
| Water Positive for *Ny. darlingi* | 95.42% | 0.06% |  | 94.92% | 0.06% |
| Water Negative for *Ny. darlingi* | 77.85% | 0.20% |  | 97.60% | 0.05% |
| **Approach 3** |  |  |  |  |  |
| Water Positive for *Ny. darlingi* | 96.99% | 0.05% |  | 96.90% | 0.05% |
| Water Negative for *Ny. darlingi* | 96.54% | 0.05% |  | 96.70% | 0.05% |
