# Supplementary material for: High-accuracy detection of malaria vector larval habitats using drone-based multispectral imagery
Source: PLoS Negl Trop Dis. 2019 Jan 17;13(1):e0007105. doi: 10.1371/journal.pntd.0007105 (PMC6353212; doi:10.1371/journal.pntd.0007105)
Supplement: S3 Table — (DOCX) [file pntd.0007105.s004.docx]

# Supplementary Table 3: Producer and Consumer accuracies of non-random groups for approach 1, approach 2 and approach 3.

|  | **Consumer Accuracy** | | | |  | **Producer Accuracy** | | | |
| --- | --- | --- | --- | --- | --- | --- | --- | --- | --- |
| **Classes** | **Visto Bueno** | **Libertad** | **Salvador** | **Urco Miraño** |  | **Visto Bueno** | **Libertad** | **Salvador** | **Urco Miraño** |
| **Approach 1** |  |  |  |  |  |  |  |  |  |
| Low Vegetation | 18.18% | 16.67% | 0.00% | 30.77% |  | 10.53% | 16.13% | 0.00% | 10.00% |
| High Vegetation | 60.00% | 79.44% | 41.18% | 69.49% |  | 72.86% | 77.27% | 97.22% | 92.13% |
| Bare Soil | 50.00% | 66.67% | 0.00% | 14.29% |  | 10.00% | 22.22% | 0.00% | 14.29% |
| Urban | 58.33% | 62.50% | 91.30% | 84.62% |  | 100.00% | 90.91% | 79.25% | 84.62% |
| Water Body | 100.00% | 84.85% | 86.67% | 100.00% |  | 26.67% | 100.00% | 100.00% | 75.00% |
| **Approach 2** |  |  |  |  |  |  |  |  |  |
| Low Vegetation | 10.53% | 16.13% | 0.00% | 26.67% |  | 5.26% | 16.13% | 0.00% | 10.00% |
| High Vegetation | 59.09% | 80.00% | 41.18% | 69.49% |  | 74.29% | 76.36% | 97.22% | 92.13% |
| Bare Soil | 50.00% | 66.67% | 0.00% | 14.29% |  | 10.00% | 22.22% | 0.00% | 14.29% |
| Urban | 59.57% | 60.00% | 91.30% | 84.62% |  | 100.00% | 81.82% | 79.25% | 84.62% |
| Water Positive for *Ny. darlingi* | 62.50% | 68.97% | 98.15% | 0.00% |  | 35.71% | 86.96% | 91.38% | 0.00% |
| Water Negative for *Ny. darlingi* | 100.00% | 89.74% | 67.74% | 100.00% |  | 71.43% | 92.11% | 100.00% | 100.00% |
| **Approach 3** |  |  |  |  |  |  |  |  |  |
| Water Positive for *Ny. darlingi* | 76.47% | 86.96% | 100.00% | 100.00% |  | 100.00% | 86.96% | 91.38% | 100.00% |
| Water Negative for *Ny. darlingi* | 100.00% | 92.11% | 80.77% | 100.00% |  | 71.43% | 92.11% | 100.00% | 100.00% |
