# Supplementary figures and images for: High-accuracy detection of malaria vector larval habitats using drone-based multispectral imagery
Source: PLoS Negl Trop Dis. 2019 Jan 17;13(1):e0007105. doi: 10.1371/journal.pntd.0007105 (PMC6353212; doi:10.1371/journal.pntd.0007105)

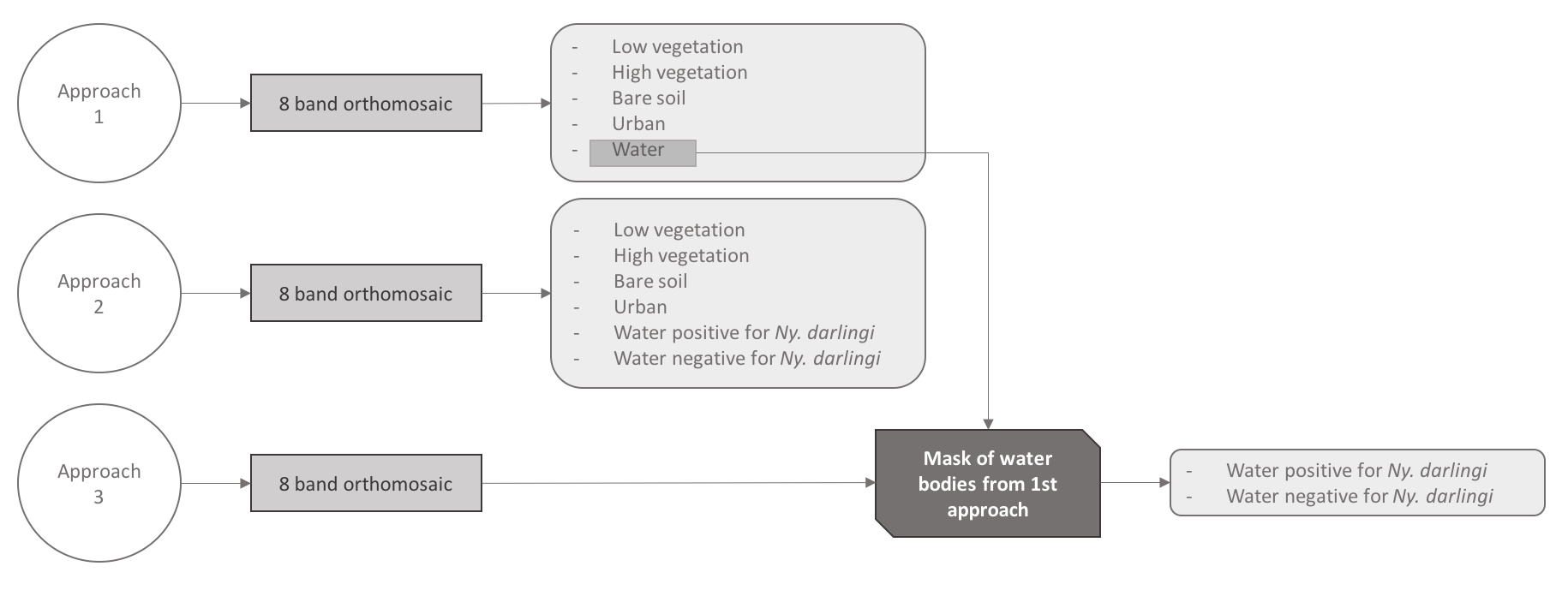

Supplement: S1 Fig — (TIF) [file pntd.0007105.s005.tif]

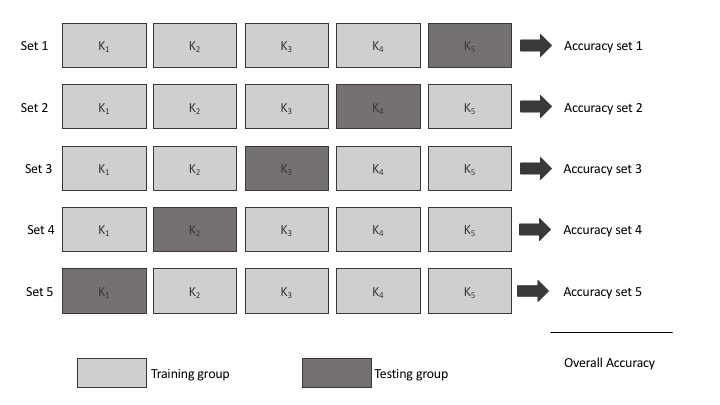

Supplement: S2 Fig — (TIF) [file pntd.0007105.s006.tif]

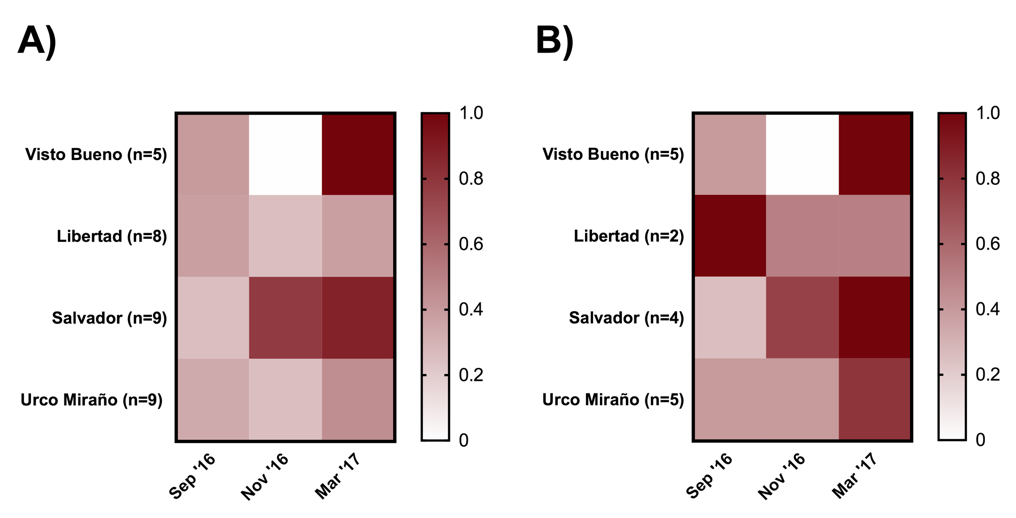

Supplement: S3 Fig — a) All water bodies sampled and b) 16 water bodies selected for multispectral mapping. (TIF) [file pntd.0007105.s007.tif]

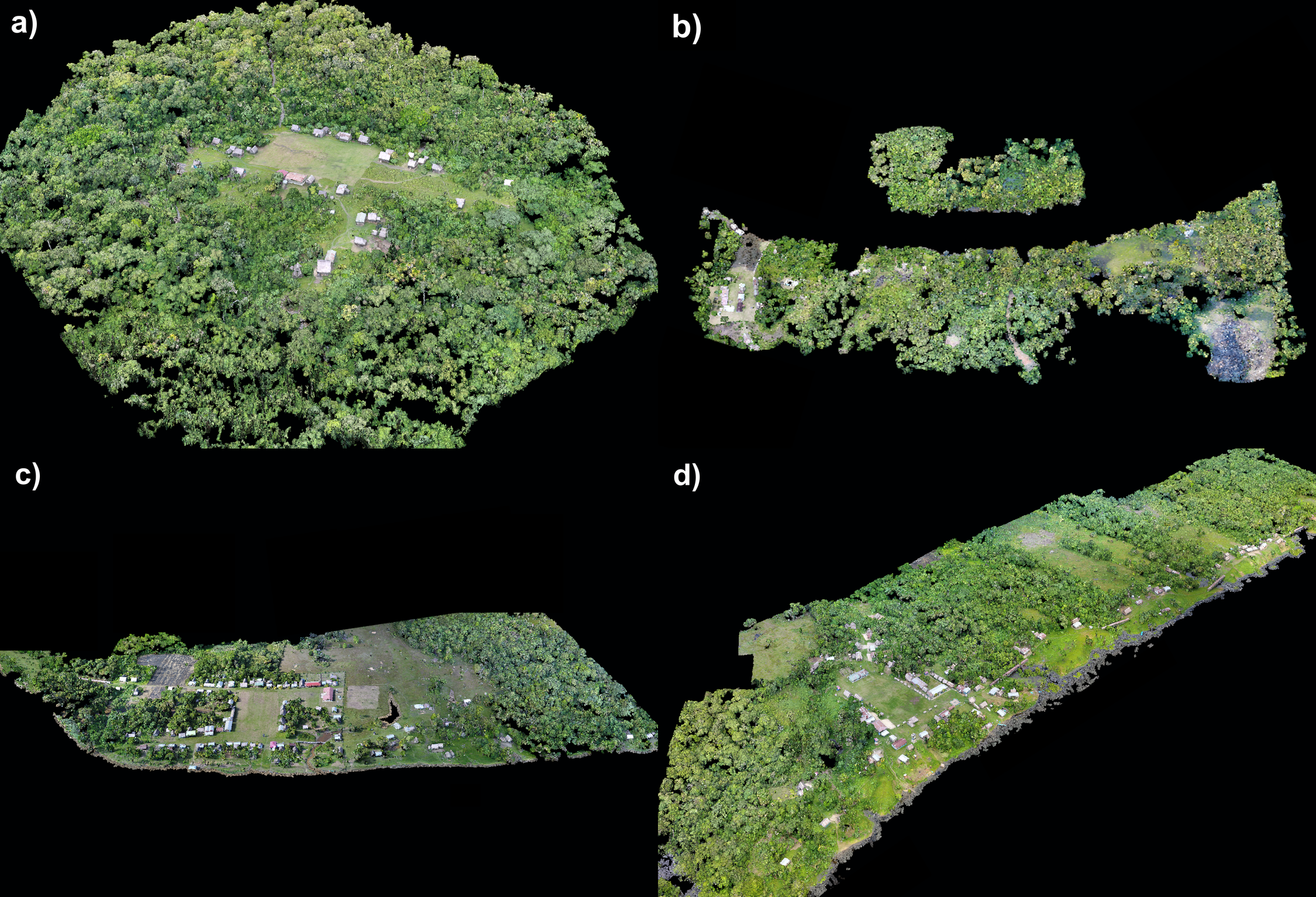

Supplement: S4 Fig — 3D models were constructed and mapped in AgiSoft Photoscan Pro (https://www.agisoft.com)based on drone imagery. (TIF) [file pntd.0007105.s008.tif]

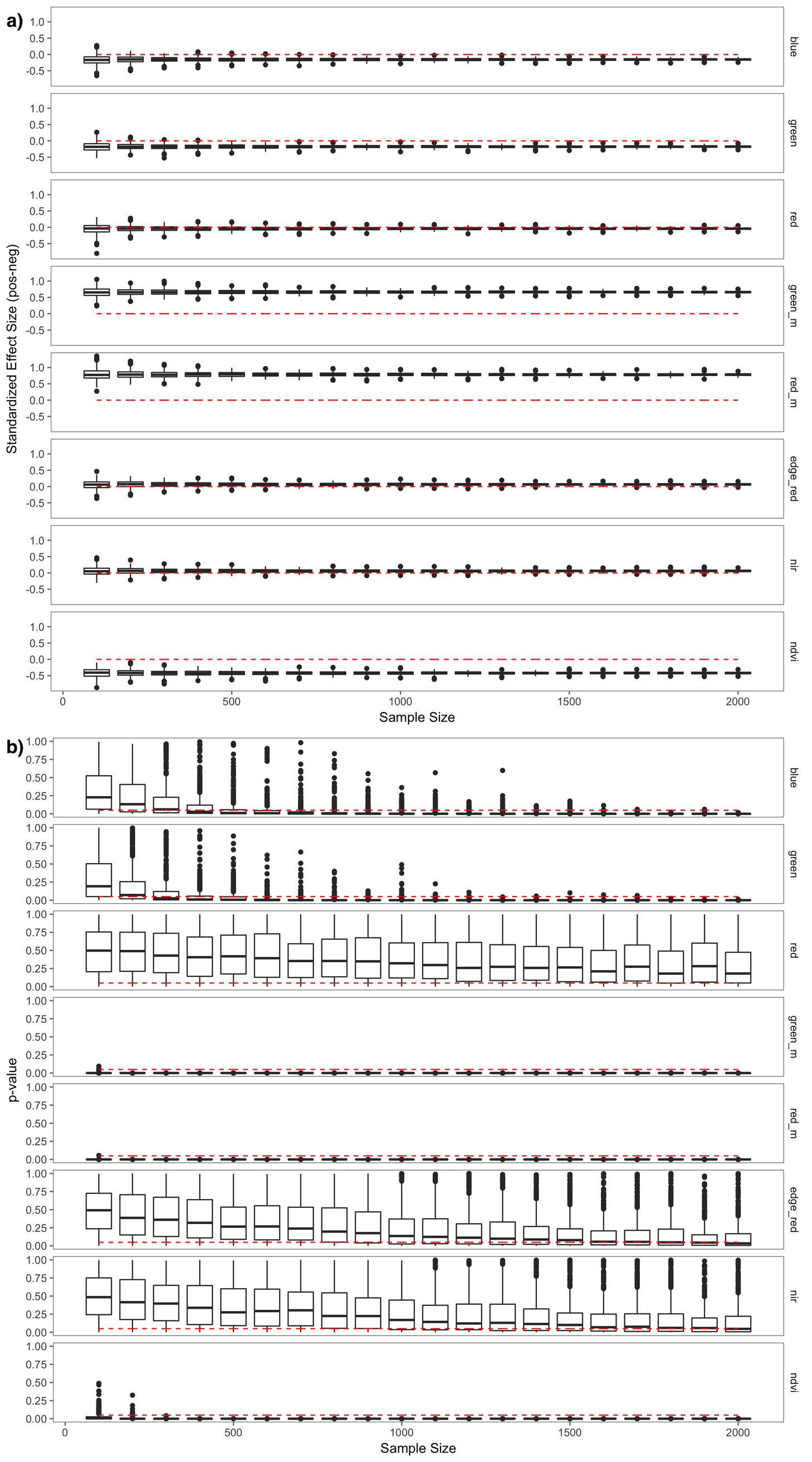

Supplement: S5 Fig — a) Standardized effect size and b) p-value as a function of sample size. (TIF) [file pntd.0007105.s009.tif]

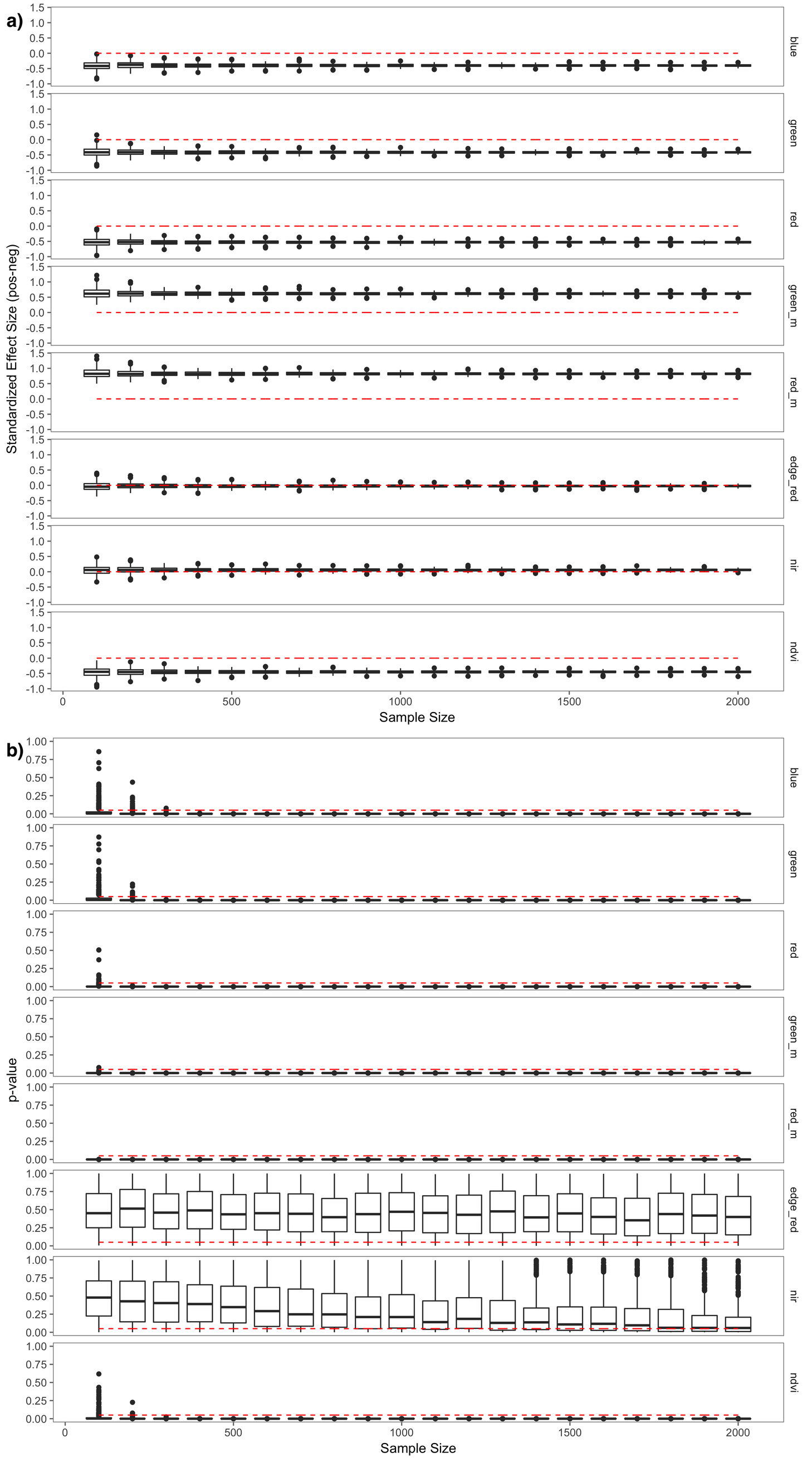

Supplement: S6 Fig — a) Standardized effect size and b) p-value as a function of sample size. (TIF) [file pntd.0007105.s010.tif]
