# Supplementary material for: High-accuracy detection of malaria vector larval habitats using drone-based multispectral imagery
Source: PLoS Negl Trop Dis. 2019 Jan 17;13(1):e0007105. doi: 10.1371/journal.pntd.0007105 (PMC6353212; doi:10.1371/journal.pntd.0007105)
Supplement: S1 Data — (DOCX) [file pntd.0007105.s011.docx]

# S1 DATA. DATA AND CODE AVAILABILITY

**Dataset 1:** Metadata regarding number of visits per water body and the definition of on-water classes for GEE random forest classification available at <https://doi.org/10.6084/m9.figshare.7041392.v1>

**Dataset 2:** Training and test sets for random forest classification in Google Earth Engine available at <https://doi.org/10.6084/m9.figshare.7053284.v1>

**Dataset 3:** Orthomosaics input, Classification output and Code example for random forest classification, and overall, producer and consumer accuracy in Google Earth Engine repository available at <https://code.earthengine.google.com/f07563d1be139080cf517d6ab950d912>
